# Pancreatic Cancer and Diabetes: Insights, Hypotheses, and Next Steps

**DOI:** 10.3390/ijms262110245

**Published:** 2025-10-22

**Authors:** Jessica L. E. Hill, Thomas G. Hill, Dominique Parslow, David J. Hill

**Affiliations:** 1 Keele Medical School, University of Keele, Newcastle ST5 5BG, UK; y0w14@students.keele.ac.uk; 2 Oxford Centre for Diabetes, Endocrinology and Metabolism, University of Oxford, Oxford OX3 7LE, UK; thomas.hill2@ocdem.ox.ac.uk; 3 University Hospitals Plymouth NHS Trust, Derriford Road, Plymouth PL6 8DH, UK; dominiqueparslow@nhs.net; 4 Lawson Research Institute, St. Joseph Health Care, London, ON N6A 4V2, Canada; 5 Departments of Medicine, Physiology and Pharmacology, Western University, London, ON N6A 3K7, Canada

**Keywords:** PDAC, diabetes, new-onset diabetes, type 3c diabetes

## Abstract

Pancreatic ductal adenocarcinoma (PDAC) is frequently associated with new-onset diabetes (NOD) in adults aged ≥ 50 years. Accordingly, NOD may serve as an early clinical marker for PDAC, although the causal links remain incompletely defined. This review synthesises clinical and experimental evidence into an islet-centric view in which tumour-derived signals and microenvironmental changes impair β-cell insulin secretion and disrupt α- and δ-cell regulation. We distinguish findings established in PDAC from mechanisms inferred from islet physiology and systemic metabolism. Key uncertainties include the timing of systemic versus local drivers, the clinical relevance of tumour-derived signals in driving dysglycaemia, the status of endocrine cell signalling and endocrine–exocrine crosstalk, and the effects of microvascular and matrix changes on endocrine–vascular exchange. This synthesis highlights mechanisms that remain incompletely defined and prioritises areas for further research.

## 1. Introduction

Pancreatic ductal adenocarcinoma (PDAC)-associated diabetes mellitus (type 3c diabetes; hereafter PDAC-DM) is often overlooked in research due to the immediacy of pancreatic cancer care. In day-to-day practice, the lethality of PDAC pushes hyperglycaemia from type 3c diabetes to the background, and few clinicians specialise at the intersection of oncology and diabetology [1,2]. As a result, PDAC-DM has often been assumed to pathologically mirror type 2 diabetes, and the field has been fractured around inconsistent disease descriptions—glycaemic dysregulation, glycaemic dysfunction, insulin-sensitive, insulin-deficient, new-onset diabetes (NOD), or type 3c diabetes—without a shared, mechanistic vocabulary [3,4,5]. This disciplinary gap has limited visibility at major international conferences and hindered consensus on endpoints, cohorts, and study timing. Yet the clinical pattern suggests a tumour-driven component that warrants focused attention: dysglycaemia can precede tumour detection and sometimes improves after resection [6]. These features are more consistent with a paraneoplastic process than with conventional type 2 diabetes alone. Mechanistically, emerging data support PDAC-DM not as a single-defect disorder but as tumour-influenced, multi-compartment dysregulation with an incompletely defined pathological trajectory whose relative drivers likely shift over time [7,8].

Against this backdrop of terminology inconsistency and paraneoplastic-leaning clinical patterns, this review has two aims. First, we synthesise human evidence that PDAC influences glucose control, distinguishing what is well supported from what remains preliminary. Second, we offer a hypothesis-driven framework that leverages decades of islet physiology and PDAC stromal biology to propose testable mechanisms—spanning cell-intrinsic pathways, paracrine circuits, microvasculature, and extracellular matrix (ECM) mechanics. Throughout, we use ‘PDAC-DM’ to describe the tumour-influenced syndrome, ‘NOD’ to describe incident diabetes within 36 months of PDAC diagnosis, and ‘dysglycaemia’ to describe glucose abnormalities.

## 2. Epidemiology, Clinicopathologic Risk, Perioperative Therapy, and Metabolic Surveillance in PDAC

### 2.1. Overview

This section synthesises population epidemiology, clinicopathologic risk features, perioperative therapy, metabolic trajectories, and practical surveillance signals to guide care in PDAC and the understanding of PDAC-DM.

### 2.2. Recent Trends in Epidemiology

At the population level, diabetes is present in more than 40% of patients at PDAC diagnosis, and approximately one third meet the criteria for NOD within the 6–36 months preceding cancer detection [6,9,10,11,12]. Large cohort studies and electronic health record (EHR)-based analyses have strengthened the evidence for a two-way association between PDAC and diabetes while addressing confounding and reverse causation. In population-based analyses, people who develop NOD after age 50 years have a markedly higher short-term risk of PDAC, with the excess risk peaking in the first 6–12 months after diabetes diagnosis and then decreasing [6,10,12]. In a multiethnic cohort (48,995 African American and Latino participants), incident diabetes was associated with pancreatic cancer overall (age-standardised hazard ratio (HR) about 2.39, 95% CI 1.91–2.98), with a higher risk for NOD (≤3 years) compared to long-standing diabetes. HRs for NOD were 4.08 (95% CI 2.76–6.03) in Latino and 3.38 (95% CI 2.30–4.97) in African American individuals [10]. Earlier registry-based work developed clinical risk models for PDAC among patients with NOD, showing that aged ≥ 50, combined with weight loss and rapid glycaemic deterioration, substantially increases PDAC probability within two years [13]. Adjusted risk estimates for PDAC within two years of NOD commonly range from roughly threefold in unselected NOD populations to as high as six- to eightfold in higher-risk NOD (age 50 years or older, particularly within the first year), with further enrichment when a rapid HbA1c rise, unintentional weight loss, or early insulin requirements are present [12,13,14].

Hospital-based and national registry cohorts in East Asia corroborate these findings; for example, Korean claims and EHR-linked analyses show elevated standardised incidence ratios in the first year after NOD, with the risk remaining above the baseline for several years, particularly among those with rapid therapy escalation [15]. Evidence published within the last 5 years using biobank and regional cohorts has confirmed that diabetes is associated with an increased PDAC risk (multivariable hazard ratios generally in the 1.5–2.5 range), with higher point estimates for NOD and among individuals with weight loss or deteriorating glycaemia [16,17,18]. Enrichment is greatest in the first two years after diabetes onset and is amplified by clinical flags such as more than 5% weight loss, a rapid HbA1c rise, a new insulin requirement, or therapy intensification [11,14,17,19]. Overall, large-scale syntheses and population-based cohorts consistently support a temporal pattern in which the PDAC risk is highest shortly after diabetes onset and diminishes thereafter, consistent with a paraneoplastic component in most cases [19,20]. The features are summarised with suggested next steps in Box 1 to support bedside decision making.

Box 1 **Red flags for PDAC in NOD (≥50 years).**

- Diabetes diagnosed within the prior 6–36 months (with highest risk within the first 12 months).
- Unintentional weight loss ≥5% over 6–12 months.
- Rapid HbA1c rise (e.g., ≥1.5% within 3–6 months) or early insulin/therapy escalation.
- Glycaemic deterioration out of proportion to lifestyle changes.
- Action: When ≥2 features cluster, consider pancreatic imaging ±CA19-9, recognising CA19-9 limitations (e.g., cholestasis; Lewis-antigen-negative) (summarises Section 2.2 and Section 2.3).

### 2.3. The Challenges in Phenotyping PDAC-DM in the Clinical Setting

Type 3c diabetes is defined as hyperglycaemia secondary to diseases of the exocrine pancreas, including chronic pancreatitis and PDAC, and after pancreatectomy [3]. In contrast to type 2 diabetes, the pathophysiology is dominated by combined endocrine failure (e.g., deficient β-cell insulin and α-cell glucagon secretion) together with malabsorption from exocrine insufficiency and systemic inflammation. Clinically, type 3c diabetes may have features of exocrine insufficiency (steatorrhoea, bloating, fat-soluble vitamin deficiency), glycaemic lability with an increased risk of hypoglycaemia due to impaired counter-regulation, and a history of or imaging consistent with structural pancreatic disease. Autoimmune islet antibodies are typically absent, and overt insulin resistance features (e.g., acanthosis, marked hypertriglyceridaemia) are generally less prominent [3,4,21].

PDAC-DM can resemble recent-onset type 2 diabetes early on, so dynamic testing may be required to uncover low β-cell output, higher insulin clearance, and inadequate postprandial glucagon suppression (α-cell dysregulation) [8]. It may also present before frank exocrine insufficiency and then evolve as the tumour/stromal/islet pathology accrues [6]. Unlike chronic pancreatitis type 3c (which often shows α-cell deficiency and a hypoglycaemia risk), PDAC-DM has been shown to exhibit α-cell disinhibition that opposes insulin, which can further complicate classification and phenotyping [8].

**Figure 1:**
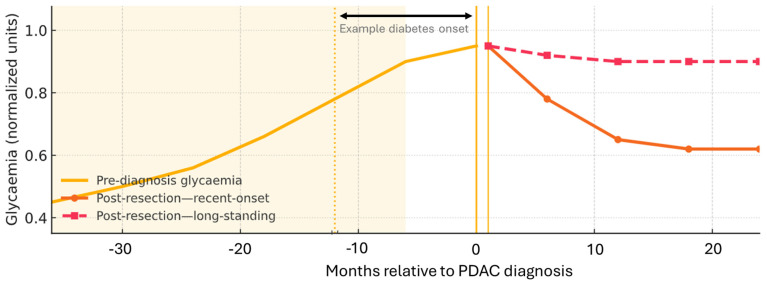
Clinical timeline of dysglycaemia relative to PDAC detection and surgery (schematic, not to scale). In some patients, NOD and weight loss appear months to years before PDAC is detected, often within a ~3-year window. Cohort and EHR studies show that the excess PDAC risk is highest in the first year after diabetes onset and then declines. After resection, glycaemia often improves for NOD, whereas long-standing diabetes shows little change. When red flags cluster in adults ≥ 50 years—e.g., >5% unintentional weight loss, rapid HbA1c rise, or early insulin requirement/therapy escalation—pancreatic imaging ± CA 19-9 should be considered (with standard CA 19-9 caveats). Summarises Section 2.2, Section 2.3 and Section 2.8.

Translating epidemiological risk to the bedside hinges on differentiating PDAC-DM from common type 2 diabetes using the timing of onset, weight trajectory, and insulin requirement. Early studies on PDAC-DM have been hindered by the insufficient removal of confounding factors for both diabetes and PDAC, such as chronic pancreatitis, smoking, and obesity [5,15]. Translating population-level signals into earlier case identification therefore depends on consistent clinical phenotyping coupled with timely imaging and carbohydrate antigen 19-9 (CA 19-9) measurement when red flags cluster. Figure 1 summarises the clinical timeline of dysglycaemia relative to PDAC detection and surgery.

### 2.4. Pancreatic Cancer Margins and Pathology Reporting

To interpret endocrine changes relevant to PDAC-DM credibly, the surgical pathology must first be standardised, because margin assessment and preanalytical handling directly shape the endocrine tissue available and the islet morphology that is preserved. Standardised specimen handling with complete identification of circumferential margins and explicit use of the 1 mm rule—defining R1 as microscopic residual tumour, where invasive carcinoma is at or within 1.0 mm of the inked margin—identifies higher true R1 rates and clarifies typical involvement at the uncinate/superior mesenteric artery and posterior planes [22,23]. Pathology reports should state the margin definition used (1 mm rule versus tumour-at-ink) and document the minimum tumour-to-margin distances, as these data inform adjuvant planning and prognosis [22,23]. Often overlooked, analyses of endocrine cells can be skewed by what is resected and sampled: the head-versus-tail location, proximity to the tumour/margins, and preanalytical factors (e.g., cautery, ischaemia/time to fixation) all influence the islet morphology and hormone staining for insulin.

### 2.5. Perineural and Intraneural Invasion (PNI/INI)

Perineural invasion (PNI) and intraneural invasion (INI) are frequent in PDAC, reflect neurotropic tumour biology, correlate with local recurrence, and should guide the adjuvant intensity [24,25,26]. Because autonomic fibres richly innervate human islets and regulate insulin and glucagon, neural invasion or injury (and the associated perineural inflammation) can perturb endocrine output and counter-regulation, linking PNI/INI to glycaemic control [27,28].

### 2.6. Adjuvant Systemic Therapy After Resection

Pathologic risk features such as the margin status, the nodal burden, and PNI/INI guide the intensity of adjuvant therapy. Randomised trials have established adjuvant chemotherapy as the standard of care after resection. CONKO-001 (gemcitabine vs. observation after resection) improved disease-free survival and later overall survival [29]. ESPAC-4 (gemcitabine plus capecitabine vs. gemcitabine) demonstrated an overall survival advantage for the combination [30]. For fit patients, modified FOLFIRINOX (oxaliplatin/irinotecan/leucovorin/infusional 5-FU) provided a substantial overall and disease-free survival benefit over gemcitabine but with increased toxicity [31]. The effects of these chemotherapy regimens on glycaemic levels in patients with PDAC-DM are limited.

### 2.7. Neoadjuvant and Perioperative Approaches

Neoadjuvant therapy offers biologic selection, earlier systemic control of micro-metastatic disease, and potential improvements in R0 rates, particularly in borderline resectable disease. This rationale is supported by consensus reviews and trial signals [32]. Contemporary programs increasingly incorporate perioperative modified FOLFIRINOX with selective chemoradiation, guided by high-quality imaging and margin-focused planning where appropriate [32,33]. When delivered, radiotherapy should follow consensus target delineation and organ-at-risk constraints with image guidance [33]. Neoadjuvant/perioperative systemic therapy, especially with steroid premedication, may transiently destabilise glucose, potentially affecting patient care for individuals with PDAC-DM; however, data are limited.

### 2.8. Diabetes Dynamics and Changes After Resection

For a subset of patients, glycaemic trajectories often improve after resection in NOD, reflecting the removal of tumour-driven dysglycaemia, although long-standing diabetes is less likely to normalise [7]. Early postoperative improvements can be transient, and close monitoring is required as adjuvant therapy, weight changes, and pancreatic exocrine insufficiency affect glucose control. Pancreatic enzyme replacement, nutrition support, and coordinated endocrine–oncology care can help to stabilise catabolism and enable adjuvant therapy delivery.

### 2.9. Deterioration and Recurrence

One analysis reported that diabetes present at diagnosis was associated with larger tumours and shorter survival after PDAC resection with adjuvant chemotherapy [34]. However, caveats include that the diabetes status was site-reported, without a uniform definition; the duration of diabetes (NOD vs. long-standing) was not captured; potential underdiagnosis/misclassification and residual confounding remained; and the analysis reflected a trial-selected, post hoc pooled cohort. Thus, further studies should determine whether NOD denotes more advanced/aggressive cancer at presentation or whether glucose dysregulation itself facilitates tumour growth.

After curative-intent pancreatectomy, relapse is common. Large series and trial analyses describe distinct patterns and timing (earlier liver vs. later lung relapse) and identify pathologic/bioclinical predictors—notably the nodal burden (e.g., lymph node ratio/N stage), tumour grade, and margin status—as correlates of the recurrence risk and site [35]. For markers of relapse, multiple cohorts show that a serial rise in CA 19-9 (a tumour marker in PDAC) can precede radiologic detection by weeks to months and yield moderate diagnostic performance [36,37,38]. Importantly, population-level analyses do not show a broad increase in recurrence risk attributable to modern glucose-lowering therapies, and evaluations should be guided by patient-level clinical deterioration rather than the medication class alone [39].

### 2.10. Therapeutics: Metformin, Glucagon-like Peptide-1 Receptor Agonists (GLP-1 RAs), and Sodium–Glucose Cotransporter 2 (SGLT2) Inhibitors

A summary of the effects of antidiabetic therapies is shown in Table 1. Metformin is a widely used oral therapy to reduce hyperglycaemia in type 2 diabetes and prediabetes. Metformin activates AMP-activated protein kinase (AMPK), suppresses hepatic gluconeogenesis, and downregulates mammalian target of rapamycin (mTOR) signalling, with antiproliferative effects reported in PDAC preclinical models [40]. Observational studies in diabetics with PDAC have described associations between metformin exposure and longer survival, but randomised and prospective trials adding metformin to standard chemotherapy have not shown a consistent survival advantage [41].

GLP-1 RAs improve glycaemic control through glucose-dependent insulin secretion, glucagon suppression, slowed gastric emptying, and enhanced satiety, yielding HbA1c reductions, weight loss, and cardiovascular benefits in type 2 diabetes. In PDAC-DM, anorexia and ongoing weight loss can constrain tolerability, yet GLP-1 RAs may stabilise glycaemia in selected patients who can maintain intake. While concerns have been raised about the impact of GLP-1 RAs on PDAC development, large cohort analyses and meta-analyses have shown no associated increased PDAC risk, and, compared with insulin, the incidence appears substantially lower and neutral relative to metformin. Meta-analyses of randomised clinical trials with GLP-1 RAs do not indicate an excess PDAC risk, aligning with contemporary assessments reporting neutral oncologic signals [42,43].

SGLT2 inhibitors lower blood glucose by promoting urinary glucose excretion, reduce heart failure events, and cause modest weight loss. Accordingly, they can be used for glycaemic control in patients with adequate oral intake and preserved renal function, with attention to risks of volume depletion and euglycaemic ketoacidosis in the context of low caloric intake. Representative SGLT2 inhibitors include empagliflozin, dapagliflozin, canagliflozin, and ertugliflozin, and agent selection should consider renal function, oral intake, and peri-operative fasting to mitigate volume depletion and euglycaemic ketoacidosis risks [44]. PDAC cells express sodium–glucose transporters (including SGLT2), and preclinical work shows functional SGLT-mediated glucose uptake that can be blocked by SGLT2 inhibitors, with growth suppression in pancreatic cancer models; however, clinical evidence for a direct anticancer effect is limited [45].

## 3. Mechanisms Linking PDAC to Dysglycaemia

### 3.1. Overview and Scope

PDAC-DM reflects two interacting processes: (i) insulin dysregulation driven by systemic insulin resistance and altered islet hormone signalling and (ii) insulin deficiency arising from β-cell stress, loss of identity, or cell loss [3,7]. We integrate tumour-intrinsic signalling programs, paraneoplastic mediators (e.g., cytokines, extracellular vesicles (EVs)), and local pancreatic niche effects (islet microvasculature, extracellular matrix, immune–stromal crosstalk) that together shape the glycaemic phenotype [3,7]. Throughout this section, we lead with PDAC-specific evidence and then identify plausible mechanisms inferred from islet biology or cancer metabolism where direct PDAC data are not yet available.

### 3.2. Tumour-Intrinsic Programmes and Metabolic Rewiring

*What is established in PDAC.* PDAC is characterised by the near-universal activation of the *KRAS* proto-oncogene GTPase (*KRAS*), with frequent loss of tumour protein p53 (TP53), cyclin-dependent kinase inhibitor 2A (CDKN2A), and SMAD family member 4 (SMAD4) [46,47]. These lesions sustain RAS–RAF–MEK–ERK (mitogen-activated protein kinase, MAPK) signalling—that is, rat sarcoma small GTPases (RAS)—activating rapidly accelerated fibrosarcoma serine/threonine kinases (RAF), which phosphorylate MAPK/ERK kinases (MEK), culminating in the activation of extracellular signal-regulated kinases (ERK). They also sustains phosphoinositide-3-kinase–protein kinase B (PI3K–AKT) signalling, the canonical pathway that integrates growth factor and insulin receptor inputs with cell growth, survival, and nutrient use. Sustained PI3K–AKT activity reprograms tumour metabolism toward an anabolic, glycolysis-biased state, diverting glucose-derived carbon into the pentose–phosphate pathway (NADPH/ribose), serine–one-carbon metabolism (nucleotide/methyl group supply), and lipid biosynthesis, thereby supporting proliferation and redox balance [48,49,50]. KRAS crosstalk with nuclear factor-κB (NF-κB) promotes inflammatory/survival programmes, while Yes-associated protein (YAP) and transcriptional coactivator with PDZ-binding motif (TAZ) integrate mechanical/mitogenic cues through TEA domain (TEAD) factors to reinforce proliferation and metabolic adaptability [51,52,53]. Loss of SMAD4 biases transforming growth factor-β (TGF-β) signalling toward desmoplasia and immune evasion [54].

*Mechanistic implications (inferred).* These oncogenic programs generate paraneoplastic mediators (cytokines and EVs) and drive stromal alterations (stiffness, hypoperfusion) that together impair PI3K–AKT-dependent insulin action in the liver and muscle and disrupt islet stimulus–secretion coupling, thereby linking core PDAC genetics to dysglycaemia [55,56].

### 3.3. Systemic Insulin Resistance: Cytokines and EVs

#### 3.3.1. Cytokines

*What is established in PDAC.* Circulating interleukin-6 (IL-6), tumour necrosis factor (TNF), and interleukin-1 (IL-1) family cytokines are elevated in pancreatic ductal adenocarcinoma (PDAC) and correlate with cachexia and adverse host metabolic profiles [55,57,58].

*What is inferred.* Across oncology and metabolic disease, IL-6 signals via Janus kinase (JAK) to activate signal transducer and activator of transcription-3 (STAT3), which induces suppressor of cytokine signalling-1/3 (SOCS1/3). SOCS1/3 bind insulin receptor substrate (IRS) adaptors and attenuate IRS–PI3K–AKT signalling, the canonical insulin effector cascade in hepatocytes and myocytes. TNF/IL-1 activate stress kinases that similarly inhibit IRS [58,59]. In hepatocytes, this favours forkhead box O1 (FoxO1)-driven gluconeogenesis, and, in skeletal muscle, it blunts AKT-dependent GLUT4 translocation and glucose uptake [60,61]. In parallel, the cytokine milieu, together with gluco-lipotoxic stress, could activate an endoplasmic reticulum (ER) stress/unfolded protein response (UPR) in β cells. By inducing SOCS3 and inhibiting insulin receptor substrate–PI3K–AKT signalling, IL-6, TNF, and IL-1 plausibly blunt insulin action in the liver and muscle and reduce β-cell stimulus–secretion coupling, thereby contributing to PDAC-DM [55,57,58,59].

#### 3.3.2. Extracellular Vesicles

*What is established in PDAC.* PDAC-derived EVs (including exosomes) reprogram host tissues. They condition the liver (premetastatic niche), remodel hepatic inflammatory and metabolic pathways, induce insulin-resistant signalling in myotubes, and, when enriched for adrenomedullin, provoke β-cell ER stress/UPR responses and suppress glucose-stimulated insulin secretion [56,62,63].

*What is inferred.* EV cargo can dampen hepatocyte AKT, upregulate gluconeogenic genes (consistent with FoxO1 derepression), and reduce muscle glucose uptake, while blunting β-cell secretion via ER stress–UPR pathways [61,62]. Because PI3K–AKT is the principal insulin pathway in the liver and muscle, EV-mediated AKT inhibition provides a direct paraneoplastic route to insulin resistance. The β-cell ER stress response supplies a parallel route to secretory failure [56,62]. Combined hepatic, muscular, and β-cell effects explain abrupt dysglycaemia and may account for the post-resection improvement observed in NOD [64].

#### 3.3.3. β-Cell Stress and Identity

*What is established (diabetes/islet biology and PDAC-relevant models).* In diabetes, chronic gluco-lipotoxic stress activates the UPR, reduces β-cell transcription factor expression (pancreatic and duodenal homeobox 1 (*PDX1*), MAF bZIP transcription factor A (*MAFA*), and NK6 homeobox 1 (*NKX6.1*)), and impairs granule priming and metabolic coupling, yielding muted first-phase insulin and damped/irregular insulin release pulsatility [64,65,66]. PDAC-derived EVs further induce β-cell ER stress and suppress insulin secretion [61].

*What is inferred.* PDAC-related inflammatory and metabolic stressors likely engage the β-cell UPR/dedifferentiation axis, producing a characteristic pattern of impaired first-phase insulin, damped insulin release pulsatility, and diminished β-cell glucose sensitivity consistent with PDAC-DM [65,66].

#### 3.3.4. Modulating Systems (Incretins, Autonomic Input, Cachexia)

*What is established.* In exocrine disease, incretin effects are reduced and can be partially recovered with enzyme replacement. Incretin hormones GLP-1 and glucose-dependent insulinotropic polypeptide (GIP) act as amplifiers of glucose-stimulated insulin secretion [67,68,69]. Cancer-associated cachexia contributes to insulin resistance and metabolic inflexibility [70].

*What is inferred.* In PDAC, these axes likely modulate rather than drive β-cell dysfunction and should be considered when interpreting glycaemic trajectories [3].

### 3.4. Fibrosis and a Model for Insulin Dysfunction

#### 3.4.1. Matrix and Perfusion

*What is established in PDAC.* The PDAC stroma is dense, hypovascular, and hyaluronan-rich, with elevated interstitial fluid pressure and microvascular compression. In preclinical models, enzymatic hyaluronan depletion with pegylated recombinant human hyaluronidase PH20 (PEGPH20) lowers the pressure, re-expands microvessels, and improves drug delivery, establishing a causal link between ECM mechanics and perfusion. However, non-selective hyaluronan targeting has not shown a survival benefit clinically [71,72,73].

*What is inferred.* Because islet hormone exchange depends on rapid capillary perfusion and short diffusion paths, elevated tissue pressure and capillary collapse are predicted to slow islet–blood exchange, which blunts first-phase insulin release and dampens pulsatility, even without overt β-cell loss. In this framework, perfusion bottlenecks and ECM expansion act as upstream physical constraints on endocrine dynamics, contributing to the early secretory phenotype of PDAC-DM [71].

#### 3.4.2. Mechanotransduction

*What is established in PDAC.* Tissue stiffening activates integrin–focal adhesion kinase (FAK) signalling, RhoA/actomyosin contractility, and YAP/transcriptional coactivator with PDZ-binding motif (TAZ) programs with *TEA* domain transcription factors (TEADs). These pathways cooperate with KRAS–ERK, mitogen-activated protein kinase (MAPK), and AKT signalling to support growth and immune exclusion. The inhibition of FAK or YAP/TEAD remodels the stroma and improves antitumour responses in preclinical PDAC models [52,53,74,75,76].

*What is inferred.* The same integrin FAK–RhoA/actomyosin→YAP/TAZ–TEAD mechanotransduction network that senses matrix stiffness in PDAC stromal/tumour cells [74,75,76,77] may alter β-cell mechanosensing at the capillary/ECM interface of islets, where integrin–FAK signalling focuses insulin release [78,79,80]. In human/murine islets, local β1-integrin activation directs insulin granule fusion toward capillaries (targeting secretion to the vascular pole), and piezo-type mechanosensitive ion channel component 1 (PIEZO1) mechanically regulates the β-cell membrane potential, Ca^2+^ influx, and insulin secretion. Thus, stromal stiffening and an altered capillary architecture plausibly slow KATP–Ca^2+^ coupling and reduce SNARE-mediated exocytosis, manifesting as lower β-cell glucose sensitivity and reduced pulsatility in PDAC-DM [78,81,82,83,84,85].

#### 3.4.3. Immune–Matrix Crosstalk

*What is established in PDAC.* Cancer-associated fibroblast (CAF) subsets, both myofibroblastic and inflammatory, deposit collagen/hyaluronan and secrete transforming growth factor-β (TGF-β), IL-6, and chemokines. Stromal C-X-C motif chemokine ligand 12 (CXCL12) retains C-X-C chemokine receptor 4 (CXCR4)-positive T cells in the stromal rim, limiting tumour entry. Disrupting CXCL12–CXCR4 enhances responses to checkpoint blockade. Impaired epithelial TGF-β restraint further biases tissues toward fibrotic, immune-excluding microenvironments [54,71,72,86,87,88,89].

*What is inferred.* CAF-driven matrix deposition and CXCL12-dependent T-cell sequestration stabilise a state of stiffness and cytokinaemia (notably IL-6 and the activation of STAT3/SOCS), which raises the interstitial pressure and sustains systemic insulin resistance (via IRS signalling through PI3K and AKT inhibition in the liver/muscle). The same immune–matrix programs that enforce immune evasion simultaneously (i) impair islet perfusion locally and (ii) increase hepatic glucose output/reduce muscle glucose uptake peripherally, which together could worsen fasting and postprandial glycaemia in PDAC [55,69].

#### 3.4.4. Islet-Adjacent Pathology

*What is established in PDAC.* Human studies on PDAC-DM demonstrate peri-islet fibrosis, thickening of the islet basement membrane, frequent islet invasion, and reduced expression of insulin (*INS*) and glucagon (*GCG*) transcripts [90,91]. A post-resection glycaemic improvement in a subset of patients with NOD supports a reversible paraneoplastic component [92,93].

*What is inferred.* Basement membrane expansion and peri-islet fibrosis increase the diffusion distance and reduce the capillary–islet exchange rates, while islet invasion perturbs the paracrine architecture (β-cell to α-cell restraint via insulin, Zn^2+^, and Gamma-aminobutyric acid (GABA) actions; δ-cell to α-cell restraint via somatostatin signalling through SSTR2 and SSTR5). These structural changes are compatible with insulin deficiency, muted first-phase insulin, damped pulsatility, and α-cell disinhibition. This could provide a tissue-level explanation for the endocrine phenotype of PDAC-DM and its reversibility after tumour removal [90,91].

## 4. Toward a Hypothesis-Driven Model of PDAC-Associated Dysglycaemia: Islet, Vascular, and ECM Mechanisms

### 4.1. Overview

**Figure 2:**
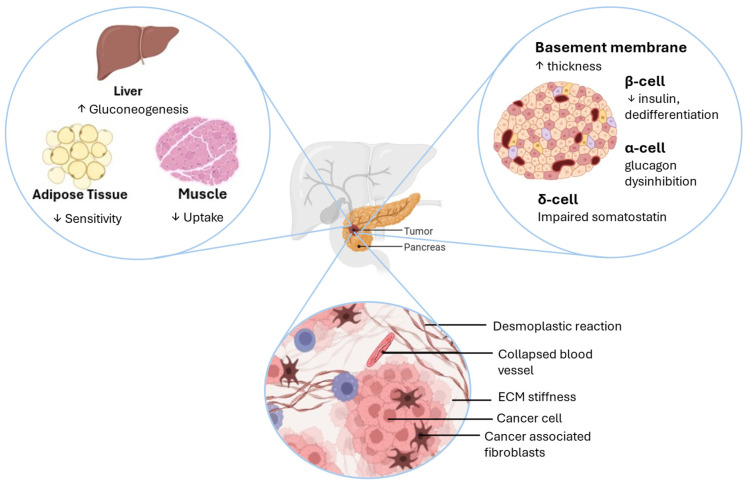
Proposed mechanisms of PDAC-DM. Illustration of a proposed framework whereby PDAC perturbs systemic and islet glucose regulation. Changes in muscle, adipose, and liver, together with loss of islet hormonal balance, may result in progressive dysglycaemia and diabetes. Tumour-derived mediators promote hepatic gluconeogenesis, impair skeletal muscle glucose uptake, and reduce adipose insulin sensitivity. Within islets, β cells exhibit reduced insulin secretion and dedifferentiation, α cells show glucagon disinhibition, and δ cells have impaired somatostatin signalling. Stromal alterations including basement membrane thickening, desmoplasia, extracellular matrix stiffening, vascular compression, and cancer-associated fibroblasts may further contribute to dysglycaemia. Arrows indicate directional increase or decrease in PDAC-DM.

Studies on endocrine cells in PDAC-DM are sparse. We address this gap by first summarising the endocrine phenotypes documented in PDAC-DM. We then outline testable mechanisms linking tumour biology to β-, α-, and δ-cell function and to the islet–vascular interface. Figure 2 shows an integrated schematic. Throughout, we distinguish findings demonstrated in PDAC from hypotheses inferred from islet physiology and cancer biology.

### 4.2. β-Cell Stimulus Secretion and Identity

Early β-cell dysfunction in PDAC-DM presents with three linked features: a muted first-phase insulin response to glucose, damped or irregular pulsatile insulin release, and reduced β-cell glucose sensitivity [94,95,96,97,98]. These changes can precede tumour detection by months to years and commonly accompany variable insulin resistance and weight loss [12,92,93]. Mechanistically, they map onto the canonical stimulus–secretion pathway, in which glucokinase-driven metabolism raises the ATP/ADP ratio, which closes the β-cell plasma ATP-sensitive K^+^ (K_ATP_) channels, leading to the depolarisation of the membrane. This membrane depolarisation subsequently leads to the opening of the Ca^2+^ channels, triggering SNARE-mediated granule exocytosis, with anaplerotic, redox, and lipid-amplifying signals further shaping the output [84,85,99,100]. In PDAC, tumour- and stroma-derived mediators, including inflammatory cytokines and EVs, plausibly hinder these steps by altering ion channel activity, vesicle priming, and metabolic coupling. However, direct in vivo confirmation in human PDAC remains limited. Moreover, exocrine insufficiency, cancer-associated weight loss, and systemic inflammation can modify the glycaemic phenotype and should be considered when interpreting early β-cell dysfunction [3,58,70].

Chronic inflammatory and metabolic stress may also induce a loss of β-cell identity by downregulating expression of maturity-associated transcription factors such as PDX1, MAFA, and NKX6.1, which will reduce insulin biosynthesis and the efficiency of stimulus–secretion coupling. This is well supported by diabetes models and is increasingly suggested for PDAC, although large human PDAC islet datasets are still sparse [45,65,66]. Taken together, a β-cell-centric view could contribute to the phenotype but presumes intact islet paracrine restraint and vascular exchange. This may occur in the early stages of PDAC before more intensive insulin therapy is required (Table 2).

### 4.3. α-Cell Disinhibition and Paracrine Control

Inappropriate glucagon secretion, especially a failure to suppress glucagon during hyperglycaemia, antagonises insulin and can blunt the early insulin response and dampen pulsatility. α-cell excitability is directly modulated by glucose through K_ATP_-dependent mechanisms, but, in intact islets, it is also strongly influenced by β- and δ-cell signals. β-cell-derived secretory products (such as insulin, zinc, and GABA_A_) and δ-cell-derived somatostatin acting via the somatostatin receptors (SSTR2/5) suppress glucagon release [85,101]. Tumour-derived cytokines and EVs, together with systemic inflammation, may perturb α-cell ion channel and receptor pathways and thus favour disinhibition, although the human PDAC-specific α-cell physiology remains under-characterised [58,63]. Given the prominence of somatostatin-mediated restraint on α-cell glucagon secretion, the extent of α-cell disinhibition in PDAC also likely depends on δ-cell function and the microenvironment that sustains it (Table 2) [85,101].

### 4.4. δ-Cell Somatostatin Gating

δ cells orchestrate the short-range gating of both α and β cells via somatostatin, thereby sharpening the onset and amplitude of insulin pulses and ensuring appropriate glucagon suppression after meals [102,103]. In PDAC-adjacent islets, several factors could undermine this gating. Inflammatory cytokines and cargo within EVs would likely reduce δ-cell excitability or secretory efficiency (especially with β-cell dysfunction), while peri-islet fibrosis and basement membrane thickening may shorten the effective range of somatostatin and altered paracrine inputs (for example, urocortin-3 signalling via corticotropin-releasing hormone receptor 2 (UCN3–CRHR2), GABA, and serotonin dynamics) may disrupt the timing of δ-cell hormonal output [104]. Direct human PDAC data that identify δ-cell defects are still limited, so these inferences rely on well-established islet physiology combined with PDAC stromal pathology. Even so, modest decrements in δ-cell function would predictably reduce the temporal precision of insulin–glucagon coordination (Table 2) [103]. Crucially, these paracrine mechanisms can only shape systemic glucose if hormone exchange with the blood is sufficiently rapid, which depends on the microvasculature and ECM, as addressed next.

### 4.5. Microvasculature, Basement Membrane, and ECM Mechanics

The desmoplastic, hypovascular stroma associated with PDAC elevates the interstitial fluid pressure, compresses microvessels, and impairs perfusion [71]. As PDAC progresses and patients require more intensive insulin therapies, peri-islet fibrosis and thickened basement membranes likely degrade oxygen delivery and slow capillary–endocrine cell exchange, both of which are known to blunt first-phase insulin and dampen pulsatility [71,72,90,105,106]. Beyond bulk flow, endothelial glycocalyx shedding, reduced fenestration, pericyte dropout, and matrix remodelling can each impede the rapid bidirectional movement of insulin, glucagon, and somatostatin needed for crisp pulsatile release and timely suppression [58,89,107]. Increased stiffness and altered integrin signalling can also modify ion channel behaviour and vesicle dynamics in endocrine cells, plausibly slowing K_ATP_–Ca^2+^ coupling and SNARE-mediated exocytosis [74,76,108,109]. While the human PDAC literature linking specific microvascular features to contemporaneous islet function is underdeveloped, the mechanistic plausibility is strong and consistent with both the stromal pathology in PDAC and known requirements for fast endocrine exchange. In short, PDAC-DM could arise from coordinated perturbations in islet-intrinsic machinery, their microenvironment, and the vascular interface (Table 3).

## 5. Gaps/Limitations and Future Directions

### 5.1. Gaps and Limitations

Important qualifiers restrict how strongly causality can be assigned. Clinical studies often define NOD inconsistently and measure biomarkers at different times relative to diagnosis or treatment, which makes the results difficult to compare across cohorts [6,7]. Common clinical confounders, including pancreatitis, pancreatic duct obstruction, cancer cachexia with IL-6-mediated metabolic effects, and treatment-related hyperglycaemia from chemotherapy corticosteroid premedication, can mimic or mask tumour-driven metabolic changes in PDAC-DM [3,55,110,111]. On the mechanistic side, direct human evidence that ties specific microvascular features (for example, loss of endothelial glycocalyx or pericyte dropout) or the basement membrane composition to contemporaneous islet function is still limited. Many influential claims are supported primarily by preclinical systems and require confirmation in PDAC patient tissues [71,72]. For cytokines and EVs, evidence remains limited in mapping the cell types that produce the key signals, what the active cargos are, and the dose–timing windows that are sufficient to reproduce discrete endocrine phenotypes in humans [56,112,113]. Finally, broad efforts to remove or shut down the tumour-supporting tissue (the stroma) have led to unexpected, sometimes harmful effects, so treatments should focus on specific stromal targets, guided by clear biological mechanisms [30,71,88].

### 5.2. Future Directions

The field now needs human-relevant experiments that prove not just associations but causation and to test whether the defects are reversible. A priority is to use organoid and tissue explant co-cultures that include islets, tumour cells, and stromal components while preserving microvessels and pericyte-like support. In these systems, defined EVs and cytokine cargos, as well as specific stromal states, should be tested for their ability to reproduce hallmark endocrine defects such as loss of first-phase insulin, damped insulin pulsatility, α-cell disinhibition, and potential reversibility. In parallel, precisely altering the ECM stiffness and integrin–cytoskeletal signalling should quantify the effects on K_ATP_–Ca^2+^ coupling, vesicle priming, and exocytosis kinetics. Finally, the detailed mapping of endothelial fenestrations, glycocalyx integrity, pericyte coverage, and the basement membrane composition in PDAC-adjacent islets, paired with indicators of insulin release through immunohistological methods, will define how the vascular structure limits endocrine dynamics. Together, these studies can separate a reversible paraneoplastic state from progressive endocrine injury and guide selective stromal and microvascular interventions that preserve islet function without undermining the antitumour efficacy.

## 6. Conclusions

PDAC-DM is best viewed as a distinct, tumour-influenced form of diabetes. A convergent model explains the signature loss of first-phase insulin and dampened pulsatility: impaired β-cell stimulus–secretion and identity, loss of normal paracrine interactions, and slowed hormone exchange from microvascular and ECM remodelling, overlaid by systemic factors (cachexia, hepatic insulin handling). Progress now hinges on standardising the terminology and timing and on human-relevant tests that move beyond association, such as organoid/explant co-cultures with vascular elements, defined cytokine/EV programmes, controlled ECM mechanics, and the direct mapping of microvascular features to islet function. The clinical integration of oncology and endocrinology will improve risk stratification for rapid-onset dysglycaemia, metabolic management during treatment, and the translation of emerging biomarkers.

## Figures and Tables

**Table 1:** Antidiabetic therapies in PDAC-associated dysglycaemia, as summarised in Section 2.10. Arrows indicate reported increase or decrease.

Class/Agent	Primary Glycaemic Effects	PDAC-Specific Oncologic Signals (Human)	Practical Selection in PDAC
Metformin	↓ Hepatic gluconeogenesis; insulin-sensitising	Observational survival associations reported; randomised add-on trials with chemotherapy have not shown consistent overall survival benefit	Useful when oral intake adequate; inexpensive
GLP-1 RAs	↑ Glucose-dependent insulin; ↓ glucagon; slows gastric emptying; weight loss	Large cohorts/meta-analyses show no confirmed increase in PDAC risk; oncologic signal neutral	Consider if weight loss is controlled and intake maintained; monitor GI adverse events
SGLT2 inhibitors	↑ Urinary glucose excretion	PDAC cells can express SGLTs (preclinical); no clinical anticancer benefit established	Consider with preserved renal function and adequate caloric intake

**Table 2:** Islet cell mechanisms in PDAC-associated dysglycaemia (β, α, δ cells). Arrows indicate directional change during PDAC-DM.

Axis	What is Documented in PDAC (Human/PDAC Models)	Mechanisms Suggested (Inferred from Islet/Oncology)
β-cell stimulus–secretion and identity	Attenuated first-phase insulin, damped/irregular pulsatility, reduced glucose sensitivity. Partial improvement after resection with NOD. PDAC-derived EVs (e.g., adrenomedullin-positive) trigger β-cell ER stress and suppress secretion (preclinical/human-informed).	Gluco-lipotoxicity → UPR → dedifferentiation (↓PDX1/MAFA/NKX6.1) impairs granule priming/coupling. Mechanosensing at the vascular face: stiff ECM → altered β1-integrin/FAK → slower Ca^2+^ dynamics/SNARE fusion.
α-cell disinhibition and paracrine control	Direct human PDAC α-cell recordings are limited. Iset physiology shows strong β→α and δ→α restraint in health.	Weakened SSTR2-mediated somatostatin restraint and loss of β-cell signals (insulin, Zn^2+^, GABA) → inappropriate glucagon; cytokines/EVs may alter α-cell ion channel signalling.
δ-cell somatostatin gating	PDAC-adjacent pancreas shows peri-islet fibrosis and thickened islet basement membrane.	Reduced δ-cell excitability/secretory efficiency and shortened diffusion radius for SST due to fibrosis/ECM; loss of temporal precision in β–α coordination.

**Table 3:** Islet–vascular interface and extracellular matrix.

Axis	What Is Documented in PDAC (Human/PDAC Models)	Mechanisms Suggested (Inferred from Islet/Oncology)
Microvasculature and perfusion	Desmoplasia, hyaluronan-rich stroma, elevated interstitial pressure, compressed vessels. Hyaluronan depletion lowers pressure and re-expands microvessels in models (no proven survival benefits clinically). Islet invasion frequent; islet basement membrane thickening documented.	Slower islet–blood exchange blunts first-phase insulin and dampens pulsatility. Pericyte dysfunction and endothelial glycocalyx loss further impede rapid hormone flux.
ECM stiffness and mechanotransduction	Matrix stiffening activates integrin–FAK and YAP/TAZ–TEAD; intersects with KRAS–ERK/PI3K–AKT; FAK/YAP inhibition remodels stroma.	Stiff ECM and altered β1-integrin/FAK at the β-cell vascular face slow exocytosis kinetics; predicts reduced glucose sensitivity and flattened pulses.
Cross-cutting paraneoplastic mediators	Cytokinaemia (IL-6/TNF/IL-1 family); EV cargo (including adrenomedullin) alters β-cell secretion and host insulin signalling.	SOCS-mediated IRS blockade (insulin resistance) + EV-induced β-cell ER-stress; additive with mechanical/vascular constraints.

## Data Availability

No new data were created or analysed in this study. Data sharing is not applicable to this article.

## Abbreviations

The following abbreviations are used in this manuscript:

AKT	Protein kinase B
AMP	Adenosine monophosphate
ATP	Adenosine triphosphate
ATP/ADP	ATP to ADP ratio
CA19-9	Carbohydrate antigen 19-9
CAF	Cancer-associated fibroblast
CD8	Cluster of Differentiation 8 (cytotoxic T cell)
CDKN2A	Cyclin-dependent kinase inhibitor 2A
CI	Confidence interval
CONKO	Charité Onkologie trial group (e.g., CONKO-001)
CXCL12	C-X-C motif chemokine ligand 12
CXCR4	C-X-C chemokine receptor 4
ECM	Extracellular matrix
EHR	Electronic health record
ER	Endoplasmic reticulum
ERK/MAPK	Extracellular signal-regulated kinase/mitogen-activated protein kinase pathway
ESPAC	European Study Group for Pancreatic Cancer
EV	Extracellular vesicle
FAK	Focal adhesion kinase
FOLFIRINOX	FOLFIRINOX regimen variant (oxaliplatin, irinotecan, leucovorin, 5-FU)
FU	Fluorouracil (5-FU)
GABA	Gamma-aminobutyric acid
GCG	Glucagon (gene/protein)
GIP	Glucose-dependent insulinotropic polypeptide
GLP-1 RA	Glucagon-like peptide-1 receptor agonist
HR	Hazard ratio
IL	Interleukin
IL-1	Interleukin-1
IL-6	Interleukin-6
INI	Intraneural invasion
INS	Insulin
IRS	Insulin receptor substrate
JAK	Janus kinase
KATP	Potassium ATP
KRAS	KRAS proto-oncogene, GTPase
MAFA	Beta-cell transcription factor MAFA
MAPK	Mitogen-activated protein kinase
NADPH	Nicotinamide adenine dinucleotide phosphate (reduced)
NKX6	NKX6 homeobox (e.g., NKX6.1) transcription factor
NOD	New-onset diabetes
PDAC	Pancreatic ductal adenocarcinoma
PDX1	Pancreatic and duodenal homeobox 1
PEGPH20	Pegylated recombinant human hyaluronidase PH20
PH20	Hyaluronidase PH20
PI3K	Phosphatidylinositol 3-kinase
PIEZO1	Piezo-type mechanosensitive ion channel component 1
PNI	Perineural invasion
PNI/INI	Perineural/intraneural invasion
SGLT2	Sodium–-glucose cotransporter 2
SMAD4	Mothers against decapentaplegic homolog 4
SNARE	Soluble NSF attachment protein receptor
SOCS	Suppressor of cytokine signallingubstrate
SST	Somatostatin
SSTR2	Somatostatin receptor 2
STAT3	Signal transducer and activator of transcription 3
TAZ	Transcriptional co-activator with PDZ-binding motif
TEAD	TEA domain family member
TGF	Transforming growth factor
TNFTGF	Tumour necrosis factorTransforming growth factor
TP53TNF	Tumour protein p53Tumour necrosis factor
UCN3-CRHR2TP53	Urocortin-3 and corticotropin-releasing hormone receptor-2 axisTumour protein p53
UPRUCN3-CRHR2	Unfolded protein responseUrocortin-3 and corticotropin-releasing hormone receptor-2 axis
YAPUPR	Yes-associated proteinUnfolded protein response
YAP	Yes-associated protein
